# Asymptomatic oesophageal stent fracture 21 months after insertion

**DOI:** 10.1016/j.radcr.2025.03.020

**Published:** 2025-03-29

**Authors:** Islam Noaman, John Gilmour, Andrew Baird

**Affiliations:** a Southeast of Scotland Radiology Training Program, Edinburgh, Scotland, United Kingdom; b Ain Shams University, Cairo, Egypt; c Clinical Radiology Department, Western General Hospital, Edinburgh, Scotland

**Keywords:** Stent, Oesophagus, Fracture, Palliative, Radiology

## Abstract

Oesophageal stenting is frequently used as a measure of palliative treatment in oesophageal cancer for the relief of dysphagia . With the advent of new modalities of immunotherapy used concurrently with conventional chemotherapy, the survival of patients with oesophageal cancer being treated with palliative intent has lengthened considerably. Consequently, there is higher likelihood of these patients experiencing stent related complications and surviving with them for longer periods of time. In this case report, we discuss a patient who was diagnosed with stent fracture 21 months after initial stent insertion.

## Introduction

Between 2015 and 2018, the UK recorded over 9000 new diagnoses of oesophageal cancer, resulting in approximately 8000 deaths annually as a result. The majority of cases and subsequent mortalities are in low and middle income countries [1].

Dysphagia remains the primary symptom for which patients with advanced disease need intervention [2].

Patients’ overall survival on palliative treatment for oesophageal cancer has lengthened considerably. The Trastuzumb trial demonstrated that the use of Trastuzumab combined with chemotherapy lengthened patients’ median survival to 13.8 months [3].

As a result, instances of stent complications are becoming a lot more commonplace, with nearly two thirds of patients suffering from a stent related complications within 6 months of placement [4].

In this case report, we describe a patient who, after experiencing good survival on palliative chemotherapy, suffered from a stent fracture that was completely asymptomatic and incidentally diagnosed.

## Case presentation

The patient is a 64-year-old lady who had presented to her general practitioner with a history of dysphagia which was initially attributed to reflux from the use of nonsteroidal anti-inflammatory medication. Conservative treatment with proton pump inhibitors failed to produce any improvement, and the dysphagia progressed to be more to solid foods. There was no significant weight loss and no other symptoms of note. Her prior medical history was significant for a Hartmann's procedure for perforated diverticular disease a few months before her first presentation with dysphagia, hypertension, and a transient ischemic attack (TIA).

An urgent referral was made for an urgent upper GIT endoscopy, which demonstrated an ulcerating mass roughly 32cm from the incisors, with concurrent stricturing of the oesophagus at that site.

**Fig. 1:**
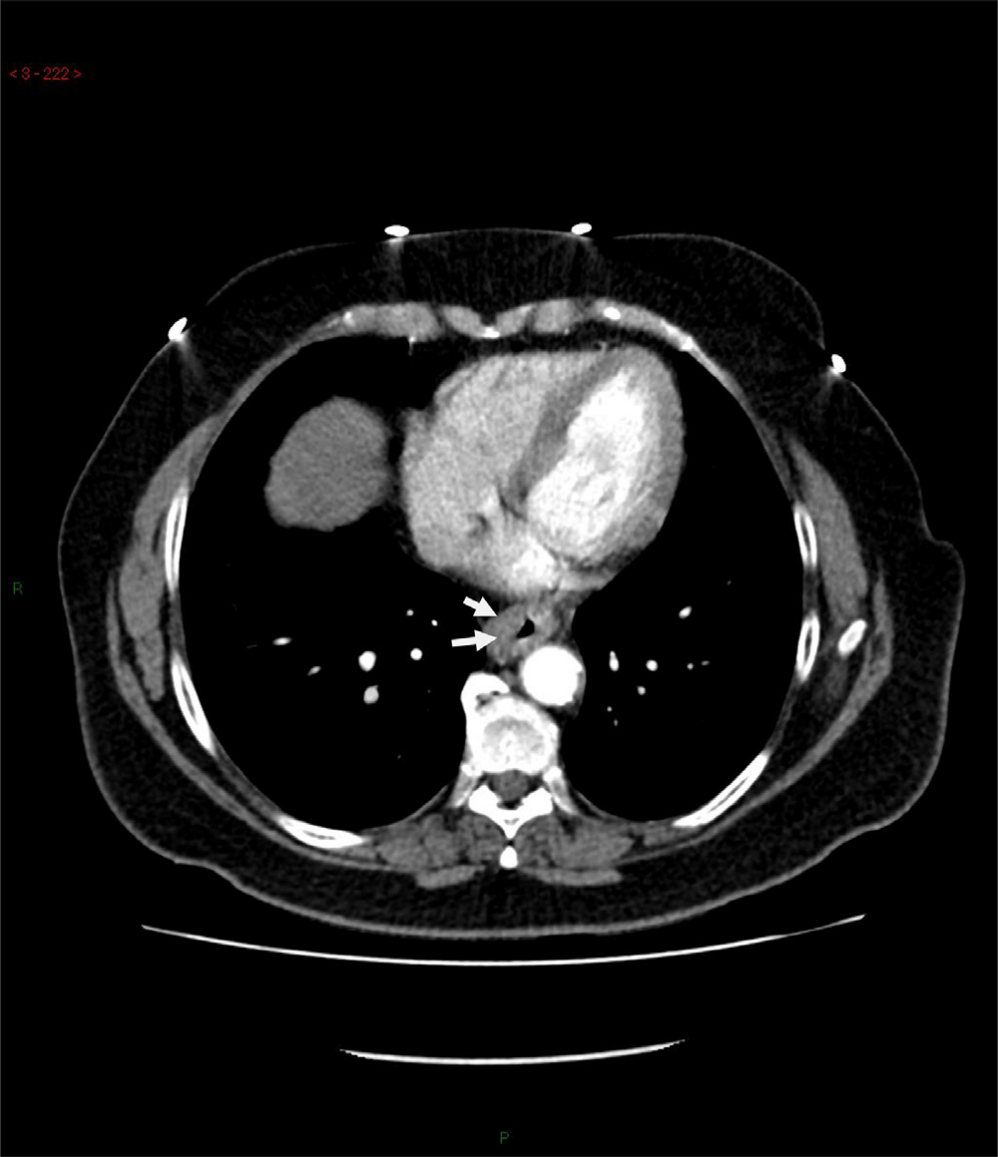
CT of the chest demonstrating thickening of the oesophagus (white arrows).

**Fig. 2:**
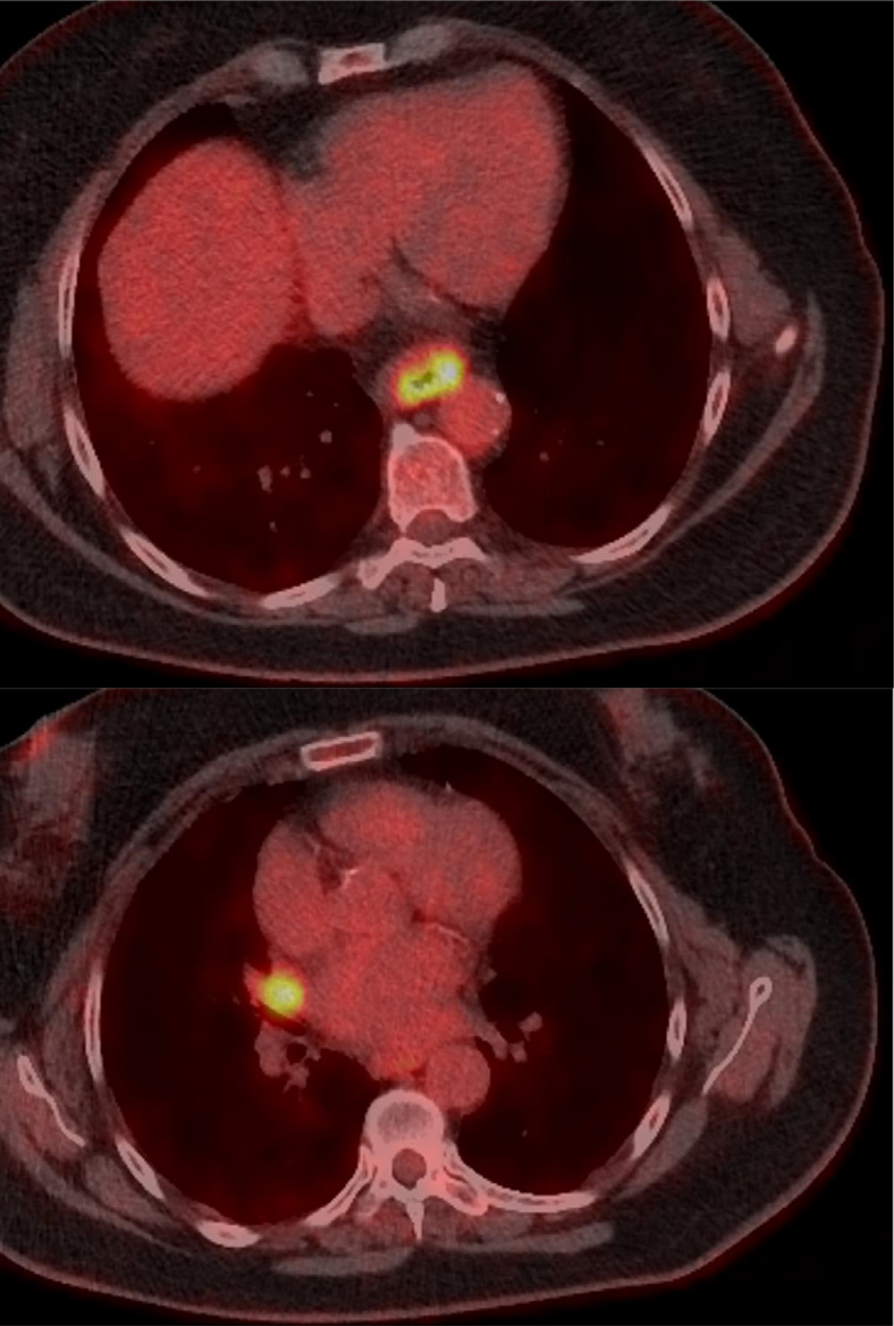
PET CT scan demonstrating uptake within the oesophageal mass (top) and the hilar mass (bottom).

**Fig. 3:**
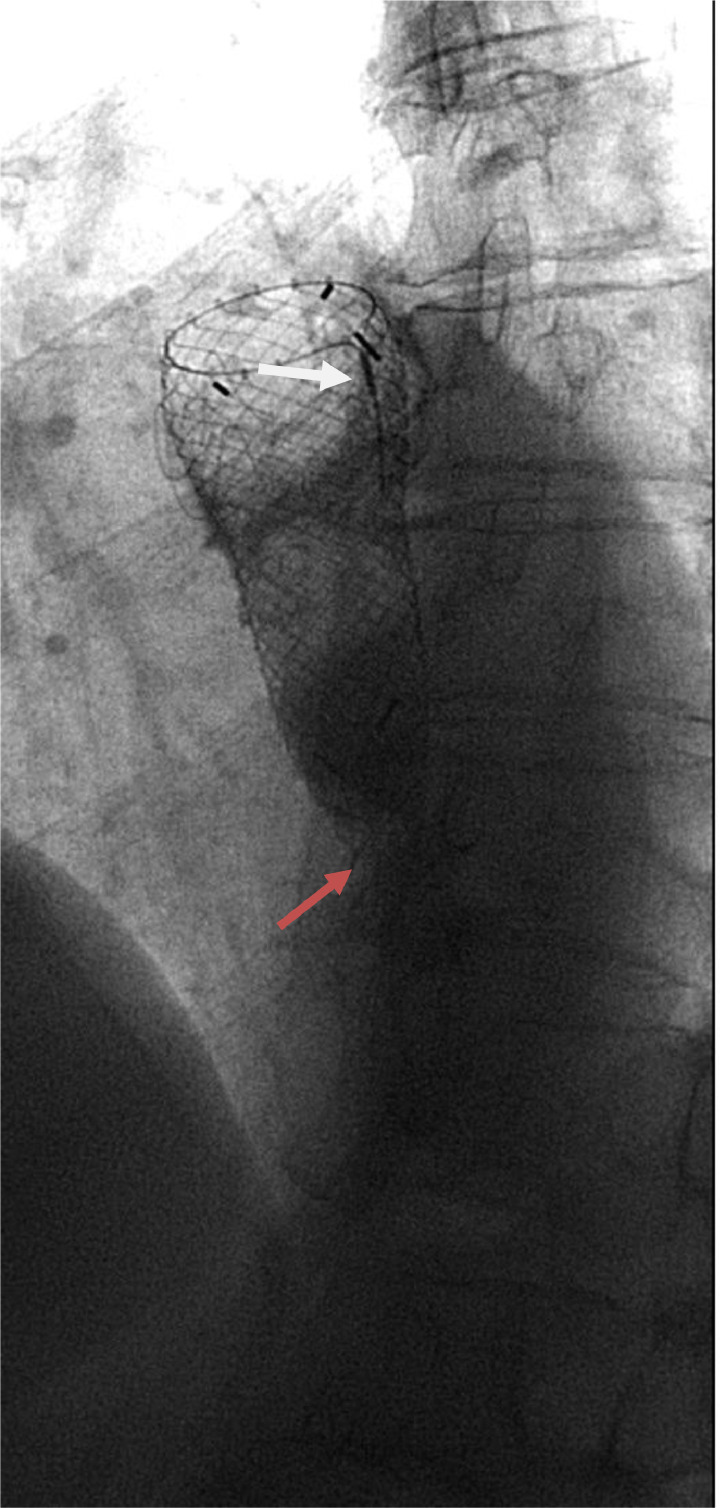
water soluble contrast swallow demonstrating intact radio-opaque markers at the top (white arrow) and missing radio-opaque markers and lower end of stent which has been fractured (red arrow).

**Fig. 4:**
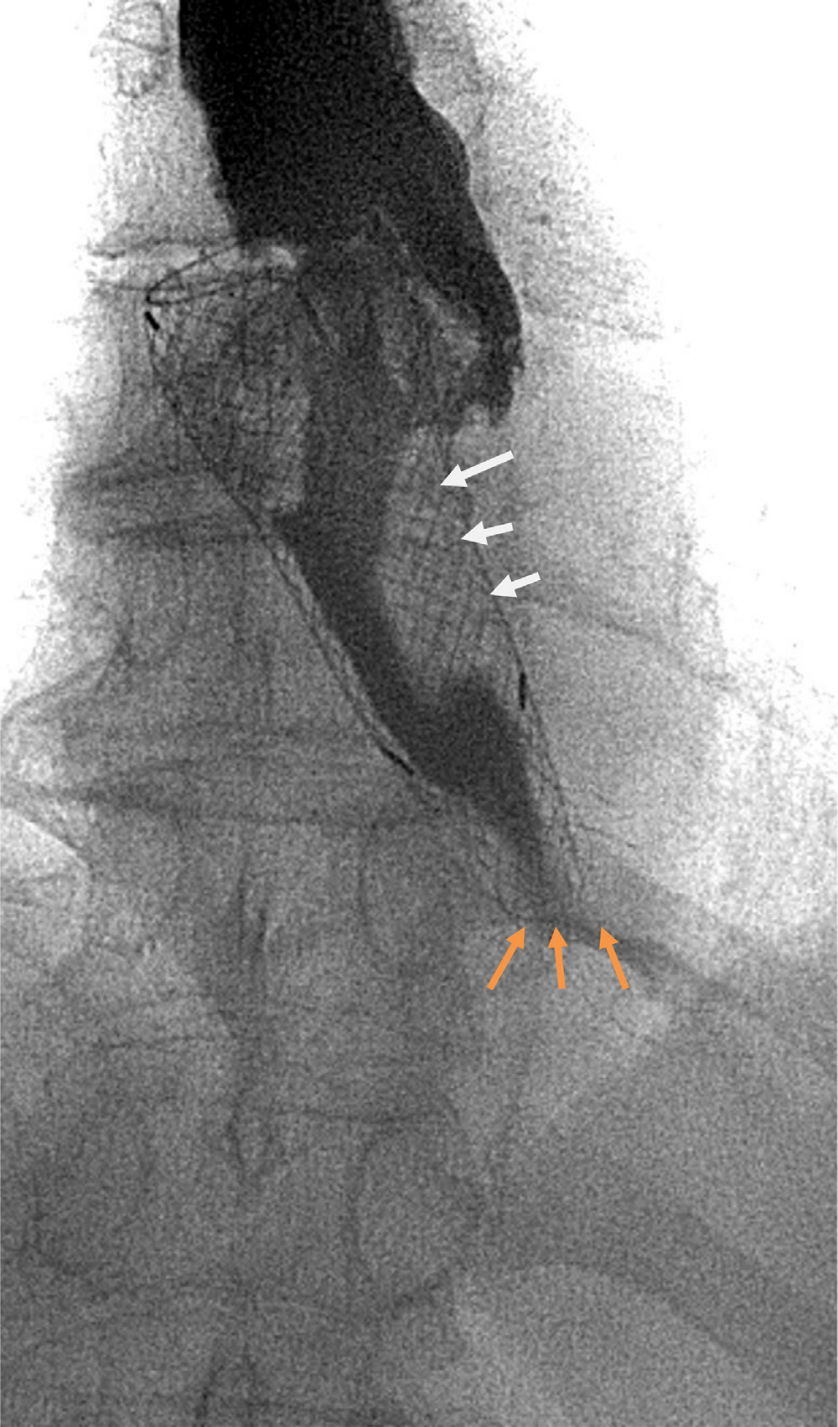
water soluble contrast swallow demonstrating filling defect (white arrows) and missing lower end of stent (orange arrows).

**Fig. 5:**
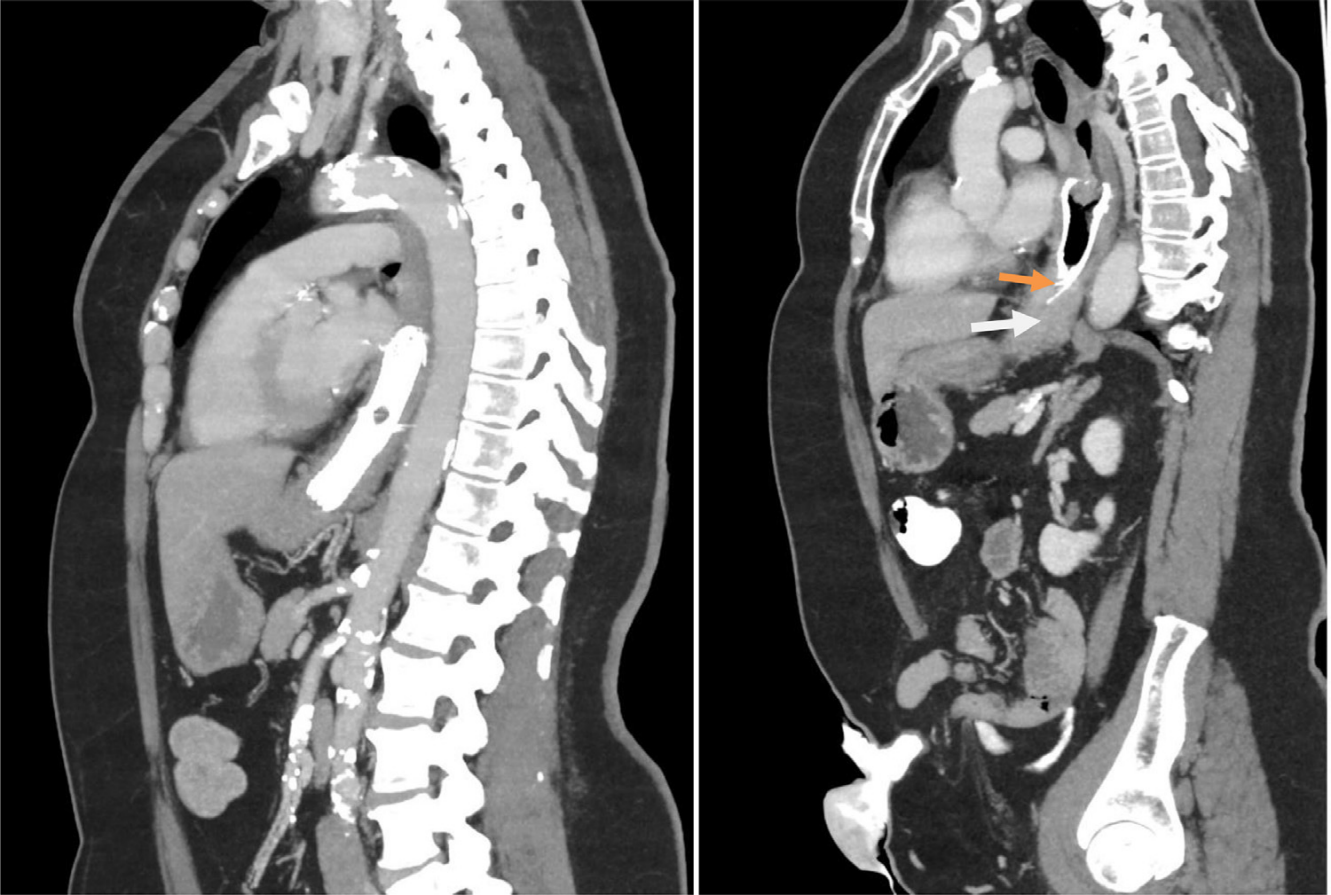
CT on the left performed 2 months prior to contrast swallow demonstrating intact stent and CT on the right immediately following contrast swallow demonstrating stent fracture (orange arrow) and tumor ingrowth (white arrow).

**Fig. 6:**
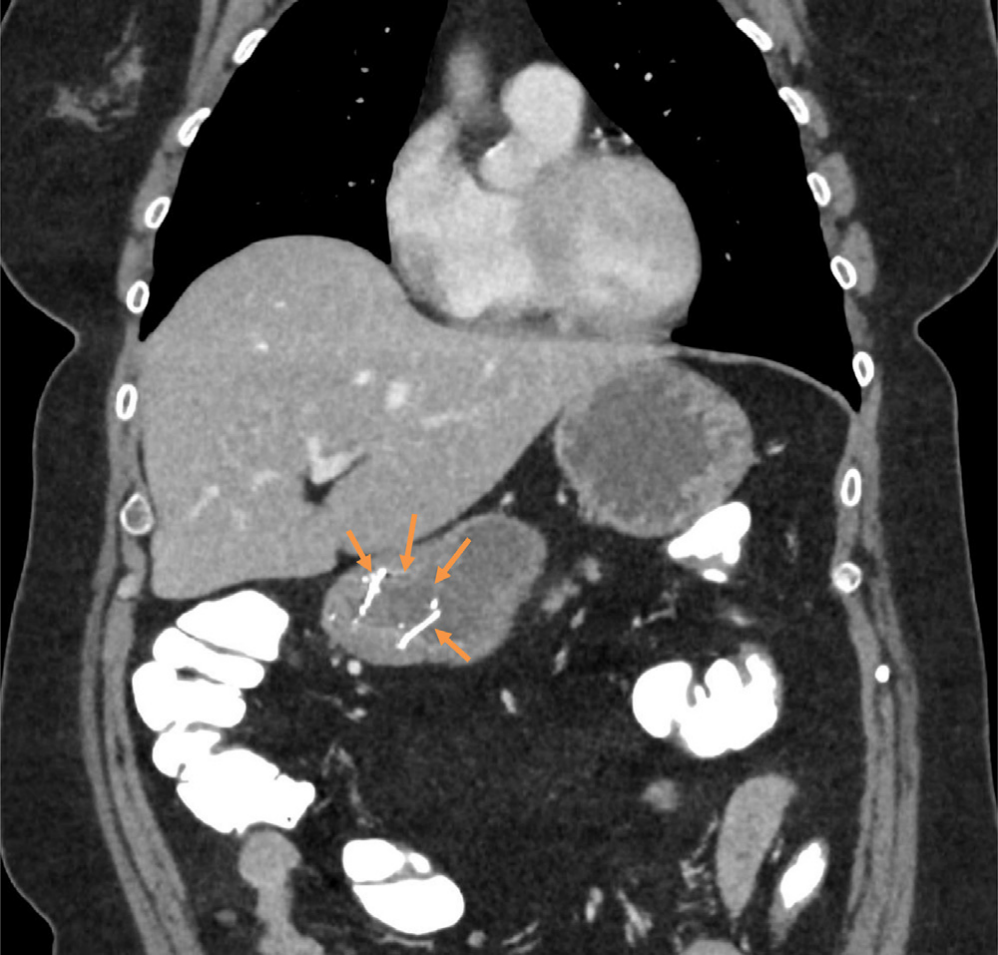
Stent fragment seen within stomach (arrows) on CT performed following contrast swallow.

Biopsy results demonstrated a poorly differentiated invasive adenocarcinoma, arising on a background of low- and high-grade dysplasia (Fig. 1, Fig. 2, Fig. 3, Fig. 4, Fig. 5, Fig. 6).

As part of her initial staging workup, she underwent a staging CT of the chest, abdomen and pelvis.

Subsequent staging PET CT demonstrated moderate uptake in the oesophageal mass but also uptake in a right hilar node/mass.

She then underwent endobronchial ultrasonography in an attempt to sample this lymph node but unfortunately was found to be too peripheral and would be risky to sample this due to proximity to the heart. In addition, the procedure had to be terminated due to an episode of severe hypoxia during the procedure.

A subsequent follow up CT of the chest with contrast demonstrated progression of the right hilar mass, and thus a consensus was reached that this was a separate hilar primary lung cancer, and the decision was made to go to palliative treatment.

As such, she underwent oesophageal stenting with a self-expandable metal stent (SEMS) approximately 21 months prior to her current presentation. She had tolerated oral intake well following that and was able to keep a steady weight.

Following her stenting procedure, she was commenced on a combination of palliative chemotherapy and immunotherapy (capecitabine, oxaliplatin and nivolumab). One month following that, the chemotherapy was discontinued due to a further ischemic event, and she was continued on immunotherapy alone.

Four days prior to her presentation, she had telephoned the oncology service mentioning a dysphagia to solids, but no other symptoms. Considering tumor ingrowth to be the most likely cause for her symptoms, a water-soluble contrast swallow was arranged.

The patient attended for the contrast swallow relatively asymptomatic; reporting only mild dysphagia from 4 days prior. Her vital signs were within normal limits (Pulse 89, BP 119/71 and temperature 36.4). The most recent labs at that point showed a hemoglobin of 130 gm/dL, white cell count of 12.5, and her renal and liver function tests were normal.

The water-soluble contrast swallow was uneventful and tolerated well by the patient.

On review of the images, the lower segment of the stent had an unusual appearance. On further inspection, the markers at the lower end of the stent were observed to be missing, the stent appeared to be shorter, and its lower end had an irregular appearance. In addition, there was a further filling defect at the upper end of the stent, indicating tumor ingrowth which was the most probable cause of her symptoms.

The fluoroscopic images were compared to the previous CT images, it was noted that the distal 2.5 cm of the stent was not visible.

The patient was transferred to the emergency department for further evaluation. Her pulse, blood pressure and temperature were all normal, and her blood tests showed a white cell count of 12.5, CRP of 34, with her liver and renal function tests all being normal.

A subsequent CT of her chest, abdomen and pelvis confirmed the stent had indeed fractured and that the distal fragment was in the pylorus, with no evidence of proximal obstruction. It also, unfortunately, confirmed the presence of tumor ingrowth which was the likely cause of her dysphagia.

The patient subsequently underwent an upper GIT endoscopy and had to have another oesophageal stent inserted, which relieved her dysphagia to good effect.

On subsequent follow up, there were neither symptoms nor complication from the fractured stent fragment in her stomach.

## Discussion

Historically, stenting to provide relief from dysphagia in the context of oesophageal cancer was reserved for advanced disease, which in turn was the most usual presentation. The use of biological therapies has caused a marked prolongation of the median survival of this cohort of patients beyond a year [5].

More significant improvement in the quality of life scores for patients with oesophageal cancer (whether on palliative or neoadjuvant treatment trajectories) can be obtained by earlier intervention [5].

Chemotherapeutic treatment of patients with oesophageal cancer was previously based on a standard regime; not considering tumor behavior. The most used regimen would be based on a platinum-5 fluoropyrimdine combination [6]. The side effects of said regime would render only 14% of patients capable of receiving chemotherapy using second line drugs according to a large United Kingdom based multi-centre study [7].

The molecular subtyping of oesophageal cancer has allowed the use of targeted therapies with less toxicity and better survival [4]. The ToGA trial, which tested the use of the Her-2 inhibitor Trastuzumab, showed a significantly improved enhanced median survival from 11.8 to 16 months for patients receiving cisplatin/5FU alone compared to patients receiving them with Trastuzumab respectively [3].

More novel therapies have reduced the toxicity from older chemotherapeutic regimens, and prolonged patient survival. As a result, almost 60% of patients receiving a stent experienced a complication related to it within 6 months [4]. Case reports of unusual stent related morbidity in the context prolonged survival range from patients needing repeated stent replacements due to recurrent stent fractures [3], to patients experiencing stent fracture following oesophageal candidiasis and embedding of the fractured stent in the oesophagus [8].

There is considerable variation in designs and materials used for the construction of oesophageal stents [9].

Within the UK, there are roughly 35 different stent designs in circulation within the NHS [4], and the lifespan of stents has been quoted as being anywhere from 1 to 6 months [4]. One of the challenges with prolonged survival in this group of patients is the production of stents with a longer lifespan [9].

Another option for the management of malignant oesophageal strictures is the use of stents loaded with radioactive brachytherapy seeds, which in certain studies was not associated with stent fracture, although this could be argued to be caused by the low overall survival of the patients in the preimmunotherapy era which was up to 11 months in this study [10].

It is also important to note that there are case reports of patients surviving long enough following immunotherapy and chemotherapy for combined lung and gastro-oesophageal cancer, to undergo a curative resection, after initial treatment with palliative intent [11].

This is a far cry from the median survival rate of patients in the initial report of the UK registry for oesophageal stenting, who in 2004, had a median survival rate of 90 days [12].

Despite improved overall survival, it is important to note that some studies quoted a re-intervention risk of 17% following oesophageal stent placement, and this rate increases to 60% at 6 months post procedure [13].

Dysphagia has been quoted to recur in roughly one third of patients following oesophageal stent insertion resulting from fracture, migration, angulation, tumor ingrowth, food bolus impaction or any combination thereof [14,15].

Although in our case there were no complications from the stent fracture per se, small bowel obstruction has been reported as a complication following oesophageal stent fracture [16]. The potential for further complications following stent fracture further adds emphasis to timely diagnosis.

In addition, with the wide variety of stents available, there is no consensus among clinicians about what constitutes optimal stent behavior in each individual use case [17], impacting stent choice and, consequently, radiological appearance.

This case, to our knowledge, shows the longest time before stent fracture was diagnosed following insertion. It is impossible to determine how long the stent was fractured for because the patient's presenting complaint was dysphagia related to her tumor ingrowth, rather than any obstructive symptoms related to migration of the fractured fragment. The range of durations found in the literature search for fracture post oesophageal stent placement was 8-40 weeks [10].

Another important note in this instance is that the first radiological investigation performed was a water-soluble contrast swallow to exclude tumor ingrowth. In this case the radiology resident failed to notice that the stent appearance was unusual in that it had a missing segment, while the experienced radiology consultant noticed that the distal radio-opaque markers of the stent were missing, and this is what prompted the CT scan that definitively identified stent fracture.

An AJR article has stated that previously that radiologists need to be familiar with various appearances of stents on contrast swallow to avoid misdiagnosing complications [18], but this case report shows that radiologists and their trainees need to be familiar with the appearance of stents in various radiological investigations, particularly in cases like this where complications may not be immediately obvious.

## Conclusion

Oesophageal stent fracture following stent placement for the palliative management of oesophageal cancer may occur at extremely variable timeframes post procedure, and these timeframes may be more prolonged given improved survival with current chemotherapeutic and immunotherapeutic regimes. This may impact subsequent clinical presentation and radiological appearances, particularly if more than 1 concurrent potential cause of post procedure dysphagia has happened as with this case. Radiologists should have a high index of suspicion when interpreting radiological investigations in this group of patients to aid in prompt diagnosis of stent related complications.

## Patient consent

I, the first author for the manuscript entitled ‘Asymptomatic oesophageal stent fracture 21 months after insertion’, declare that written and informed patient consent was obtained from the patient before submission of the manuscript, and a copy is available on request.
